# A case of babesiosis in a returning traveller

**DOI:** 10.4102/sajid.v39i1.588

**Published:** 2024-03-28

**Authors:** James W. Mac Donald, John A. Frean, John M. Ratabane, Bhavani Moodley, Karissa Mannaru, Guillaume E. Holz

**Affiliations:** 1 Department of Microbiology, Lancet Laboratories, Johannesburg, South Africa; 2 Centre for Emerging Zoonotic and Parasitic Diseases, National Institute for Communicable Diseases, Division of the National Health Laboratory Service, Johannesburg, South Africa; 3 Wits Research Institute for Malaria, Faculty of Health Sciences, University of the Witwatersrand, Johannesburg, South Africa; 4 Department of Haematology, Lancet Laboratories, Johannesburg, South Africa; 5 Specialist Physician, Private Practice, Johannesburg, South Africa

**Keywords:** babesiosis, *Babesia microti*, South Africa, malaria, *Plasmodium falciparum*

## Abstract

Human babesiosis data in Africa is scarce. The clinical presentation and parasite morphology mimics falciparum malaria infection. Diagnostic confirmation is informed by adequate history and communication with the laboratory to activate appropriate testing. This case report describes the course of a returning traveller with persisting symptoms that resolved on tailored antimicrobial therapy following prompt collaborative diagnosis.

**Contribution:**

Case highlighting overlapping characteristics of *Babesia* and malaria infection, necessitating close clinical and laboratory correlation to confirm diagnosis.

## Introduction

Babesiosis is a zoonosis caused by intraerythrocytic apicomplexan parasites.^1,2,3^ The phylum Apicomplexa is a large group of parasitic protists that also contain the parasites that cause malaria (*Plasmodium* spp.) and toxoplasmosis (*Toxoplasma gondii*).^1,4^ The primary vectors for *Babesia* spp. are ticks, and they are able to infect a number of wild and domestic animals.^1,3,5,6^ More than 100 different species of *Babesia* have been described with about six being implicated in human disease.^1,3,7^ The species most often implicated in human babesiosis in the USA is *Babesia miroti*^1^; in Europe, most cases are caused by *Babesia divergen*^3^. *Babesia microti* is endemic in northeast and upper Midwest regions of the USA and is transmitted by the tick *Ixodes scapularis* (deer tick or black-legged tick).^1,3,7^ *Ixodes scapularis* is also capable of transmitting other human pathogens such as *Anaplasma phagocytophilum, Borrelia burgdorferi, Borrelia mayonii, Borrelia miyamotoi*, Powassan virus and *Ehrlichia muris*-like organism. In the areas of the USA where more than one pathogen is present, co-infection may occur.^7^ The primary reservoir for *B. microti* is the white-footed mouse (*Peromyscus leucopus*), but other small mammals can also act as reservoirs.^7^ The various other species of *Babesia* have different vectors and hosts, depending on the geographic location, but the transmission cycle remains similar.^7^ Infection following blood transfusion and transplacental spread has also been reported.^1,7^

Human babesiosis surveillance data in Africa is scarce, but *Babesia* spp. are important veterinary pathogens.^2,6^ Differentiating babesiosis from malaria can be difficult as there is considerable overlap in the clinical presentation of both conditions.^2^ Babesias can be almost indistinguishable from *Plasmodium* spp. on peripheral blood smears. As a result of this overlap in clinical findings and diagnostic characteristics, it is likely that human babesiosis cases may be missed through misdiagnosis and underreporting.^2,5^

## Case report

A previously healthy 75-year-old male patient presented to his general practitioner (GP) in early August 2023 with a constellation of symptoms including myalgia, arthralgia, fever, nausea, anorexia, vomiting, severe abdominal pain, headaches and constipation. He had travelled to the USA 6 weeks prior to presentation. On arrival he experienced ‘flu-like symptoms and subsequently tested positive for severe acute respiratory syndrome coronavirus 2 (SARS-CoV-2). A week after arrival, the patient continued his journey to Maine, where he resided in his brother’s lake house for about 3 weeks. During his stay he partook in outdoor leisure activities. On his return to South Africa, he reported extreme fatigue and tiredness.

He was symptomatically managed by his GP for a few days. When the patient returned for review, the possibility of Lyme disease was considered (although he did not recall being bitten by ticks), and he was referred to hospital for further management.

Upon admission, the patient was acutely ill and pyrexial. Vital signs remained within acceptable limits. Physical examination revealed no overt signs of organ-specific abnormalities. The respiratory examination was unremarkable. Abdominal examination unveiled tenderness in the epigastrium, with no hepatosplenomegaly. On dermatological examination, the skin exhibited Campbell de Morgan spots. There were no mucocutaneous rashes, eschars or erythema migraines. The rest of the examination was unremarkable.

The working diagnosis encompassed several possibilities, including suspected Lyme disease related to the patient’s travel history, Gram-negative sepsis complicated by disseminated intravascular coagulopathy, and malaria. The treatment plan involved a combination of medications, including ceftriaxone, amikacin, doxycycline, pantoprazole and paracetamol, together with intravenous fluid therapy.

**FIGURE 1:**
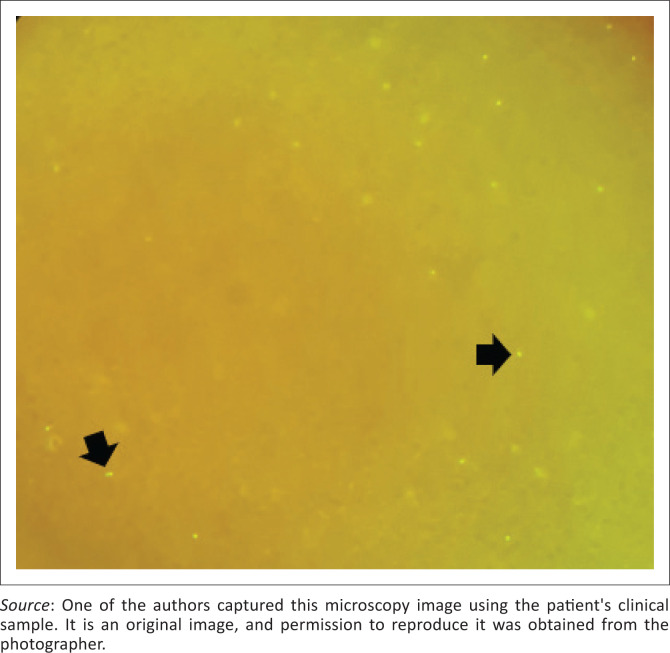
*Babesia microti* infection acquired in Maine, United States. Quantitative buffy coat microscopy of the patient’s blood sample indicating positive fluorescence signals (arrows).

The patient’s full blood and differential count reflected a mild normocytic anaemia (10.9 g/L), moderate true thrombocytopenia (54 × 10^9^/L) with normal leucocyte count (Table 1). Thin blood smear examination was initially reported as negative for malaria parasites, with negative rapid pan-malaria and *Plasmodium falciparum* antigen tests. A subsequent quantitative buffy coat (QBC) procedure was unequivocally positive for fluorescence (Figure 1) and was referred to the haematologist for diagnostic review. Quantitative buffy coat method utilises the fluorescent dye acridine orange that non-specifically stains blood parasite DNA in a capillary tube, which is then centrifuged to concentrate any parasites that are present.^8,9,10^

**TABLE 1:** Laboratory results.

Laboratory investigation	Day 0 (admission)	Day 1	Day 4	Day 25 (follow-up)	Reference range
**Haematology**					
Erythrocyte count (×10^12^/L)	3.90	3.97	3.46		4.5–6.5
Haemoglobin (g/dL)	10.9	11.2	9.7	12.5	13.8–18.8
Platelets (×10^9^/L)	54	51	59	157	150–450
Leucocyte count (×10^9^/L)	5.81	5.98	5.7		4.0–12.0
**Chemistry**					
Sodium (mmol/L)	-	135	-	142	136–145
Potassium (mmol/L)	-	4.1	-	4.8	3.5–5.1
Chloride (mmol/L)	-	105	-	-	98–107
Bicarbonate (mmol/L)	-	22	-	-	21–29
Urea (mmol/L)	7.9	8.5	-	9.5	2.9–8.2
Creatinine (mmol/L)	132	136	-	130	80–115
eGFR (CKD-EPI-mL/min/1.73 m^2^)	45	44	-	46	-
Total bilirubin (µmol/L)	25	-	-	-	3–26
Conjugated bilirubin (µmol/L)	11	-	-	-	2–7
Alkaline phosphatase (IU/L)	152	-	-	-	53–128
g-Glutamyl transferase (IU/L)	173	-	-	-	0–64
Alanine transaminase (IU/L)	80	-	-	-	<50
Aspartate transminase (IU/L)	55	-	-	-	< 50
Total protein (g/L)	70	-	-	-	60–80
Albumin (g/L)	36	-	-	-	35–50
Procalcitonin (ng/mL)	2.25	2.05	-	-	< 0.05
C-Reactive protein (mg/L)	147.4	-	102.4	-	< 5.0
**Microbiology**					
Malaria antigen	Negative	Negative	-	-	-
Malaria PCR (*P. falciparum, P. malariae, P. ovale, P. vivax*)	Not detected	-	-	-	-
*Rickettsia* PCR	Not detected	-	-	-	-
Blood cultures	No growth detected	No growth detected	-	-	-

eGFR, estimated glomerular filtration rate; CKD-EPI, The Chronic Kidney Disease Epidemiology Collaboration (CKD-EPI) equation; PCR, polymerase chain reaction.

**FIGURE 2:**
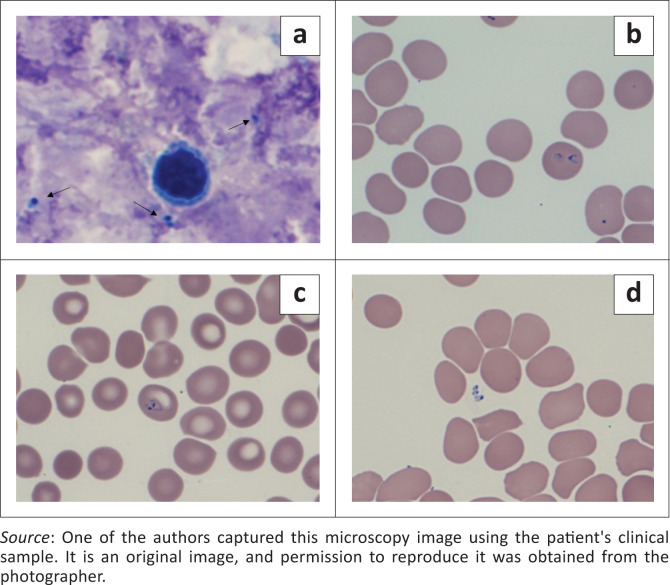
*Babesia microti* infection acquired in Maine, USA. Blood smear images, Giemsa stain, original magnification. 1000 x. (a) Thick smear showing parasites (arrows); (b, c) thin smear showing intra-erythrocytic parasites and (d) thin smear showing extra-erythrocytic parasites.

Subsequently, telephonic consultation with the treating physician confirmed specific travel history raising suspicion of a non-*Plasmodium* infection. On smear review scanty ring forms were visualised (Figure 2) and when interpreted with the clinical history, negative malaria antigen tests and positive QBC, microbiological consultation was then pursued for further epidemiological insights. Malaria polymerase chain reaction (PCR) was performed to exclude a false-negative antigen result and this was negative.

The thin smear was reviewed by microbiologists, who considered the possibility of babesiosis, given the associated travel history as well as the presence of as extra-erythrocytic parasites on microscopy. The National Institute for Communicable Diseases (NICDs) was consulted, and the consensus was that this was a likely case of travel-related babesiosis. After discussion with the treating physician ceftriaxone and amikacin were discontinued, and the patient was transitioned to clindamycin, quinine and doxycycline.

At NICD the organism was confirmed to be *B. microti* by PCR and Sanger sequencing.^11^ The parasitaemia was determined to be 0.2% on blood smear review. Lyme disease was excluded by serological testing (two-tiered serology algorithm).^12^ Co-infection with other possible tick-transmitted pathogens such as *A. phagocytophilum* and *Ehrlichia* spp. could not be excluded, and it was decided to continue further treatment with doxycycline. Two sets of blood cultures remained negative after 5 days’ incubation.

The patient’s condition gradually improved, and he was subsequently discharged after 6 days, with a directive to complete a 10-day course of doxycycline, quinine and clindamycin.

## Discussion

This case highlighted the importance of thorough history taking and good communication among the clinician, diagnostic and reference laboratories.

The diagnosis of babesiosis is most often made by identifying the organisms on a peripheral blood smear (thick and thin) stained with Giemsa or Wrights stain.^1,5^ Multiple parasites may be seen in infected erythrocytes and the appearance may resemble that of *P. falciparum*. Features such as extra-erythrocytic forms and the lack of hemozoin pigment may help distinguish babesiosis from malaria.^1,5^ The pathognomonic arrangement of merozoites in a ‘Maltese cross’ or tetrad pattern is rarely seen.^1^ Other possible laboratory findings may include anaemia, thrombocytopenia, elevated liver enzymes and deranged renal function.^1,7,13^ These laboratory features were also seen in the patient’s results (Table 1). The procalcitonin levels were raised at 2.25 ng/mL on presentation of the patient. Raised levels of procalcitonin have been reported in both human and canine cases of babesiosis.^14^ Severe malaria caused by *P. falciparum* is another non-bacterial cause for an elevated procalcitonin.^14^ While the mechanism for the elevation of procalcitonin is not completely understood in cases of babesiosis, there is interest in further investigating its potential use as a diagnostic aid in the diagnosis of human babesiosis.^14^ The procalcitonin level in the patient did respond to treatment and was measured at <0.05 ng/mL on follow up at day 25.

Clinical severity of babesiosis is largely dependent on the immune status of the patient, and the *Babesia* species involved. *Babesia microti* infections tend to be asymptomatic or mild to moderate in immunologically intact individuals. Age more than 50 years, previous splenectomy, cancer, HIV infection, haemoglobinopathy, therapeutic immunosuppression or chronic heart, lung or liver disease, predispose to more severe illness or even fatal outcome.^1,4,7^ In this patient, advanced age appears to have been a minor influence on the infection, as aided by early diagnosis and prompt treatment, serious complications were avoided.

Symptomatic babesiosis is managed with antimicrobial therapy.^1,4,8^ Treatment consists of either the combination of atovaquone plus azithromycin or quinine plus clindamycin. The duration of treatment is usually 7–10 days.^15^ The patient was managed with the latter regimen as atovaquone is mostly unavailable in South Africa.

In a malaria-endemic country, such as South Africa, it is possible to miss the diagnosis of babesiosis because of the considerable overlap with malaria in clinical and diagnostic characteristics. This could potentially lead to inappropriate treatment initiation affecting clinical outcome. To date, despite the presence of animal disease-associated babesiosis, there have been no reports of unequivocal laboratory-proven babesiosis acquired by humans in South Africa. A case of a related tick-borne piroplasm parasite infection in an HIV-positive patient in the Eastern Cape Province appears to be unique but illustrates the potential for diagnostic confusion with malaria.^16^
